# Managing Risk for Congenital Syphilis, Perth, Western Australia, Australia

**DOI:** 10.3201/eid2910.230432

**Published:** 2023-10

**Authors:** Hannah MacKenzie, Suzanne P. McEvoy, Timothy J. Ford

**Affiliations:** Western Australia Department of Health, Perth, Western Australia, Australia (H. MacKenzie, S.P. McEvoy);; Metropolitan Communicable Disease Control, Perth (S.P. McEvoy);; Perth Children’s Hospital, Perth (T.J. Ford);; University of Western Australia, Perth (T.J. Ford)

**Keywords:** congenital syphilis, communicable diseases, congenital abnormalities, sexually transmitted diseases, public health, Treponema pallidum, pregnancy, women’s health, prenatal care, health planning, disease outbreaks, bacteria, spirochete, United States, Perth, Western Australia, Australia

## Abstract

The recent resurgence of infectious syphilis across many high-income countries has been accompanied by a shift in demographics, including infections increasing among women of reproductive age. Consequently, several high-income countries are reporting increasing cases of congenital syphilis, a disease associated with a range of health and social consequences and a disease that is treatable, is preventable, and could be eliminated. To prevent congenital syphilis in the large cosmopolitan city of Perth, Western Australia, Australia, multilevel coordinated action was undertaken, including increased frequency of syphilis screening of pregnant women, workforce education and community engagement, regular interagency meetings to manage syphilis during pregnancy, use of a dynamic electronic syphilis register, use of synoptic (structured) reporting to guide management at delivery (neonatal management plans), and congenital syphilis case reviews. Other jurisdictions facing increasing syphilis cases should consider adopting these measures to reduce the risk for congenital syphilis.

Syphilis is a highly contagious sexually transmitted infection (STI) caused by *Treponema pallidum*, which has substantial short- and long-term health complications if untreated (*1*). The first line of treatment is intramuscular injection with long-acting benzathine penicillin, although reinfection can occur (*2*). Prompt contact tracing can help reduce the risk for reinfection and disease spread among sexually active persons.

Congenital syphilis is caused by transplacental *T. pallidum* transmission from mother to fetus during pregnancy (*1*,*2*). It can result in a wide spectrum of health consequences, including miscarriage, premature birth, stillbirth, low birth weight, and perinatal death, as well as brain, nerve, and organ damage (*3*–*5*). Children with congenital syphilis may face long-term disability and accompanying health, education, and societal costs. Although vertical transmission to the fetus can happen at any time during pregnancy, the risk depends on the stage of maternal syphilis and stage of gestation at which the pregnant woman acquires the infection. Untreated, the risk for vertical transmission is 70%–100% among women with primary or secondary syphilis, 40% for those with early latent disease, 10% for late latent disease, and negligible for tertiary syphilis (*5*,*6*). Characteristically, pregnant women are screened for syphilis at their first antenatal visit. Further testing may not occur during pregnancy, which may result in missed new onset illness (*7*–*9*) and substantial risk to the fetus. Fortunately, congenital syphilis is preventable and treatable (*10*) through timely detection, treatment, contact tracing, and appropriate monitoring during pregnancy.

In 2007, the World Health Organization (WHO) launched an initiative for the “elimination of congenital syphilis as a public health problem” (*11*). More recently, WHO strategic directions to reduce STIs (*12*) offer guidance for the response to rising syphilis cases: to deliver high-quality, evidence-based, people-centered services; optimize systems, sectors, and partnerships; generate and use data to drive decisions for action; engage empowered communities and civil society; and foster innovations for impact.

Of concern, cases of congenital syphilis remain elevated in many low- and middle-income countries and are resurging in several high-income countries that had previously made gains toward elimination. During the past decade, rates of infectious syphilis have increased in Australia, the United States, Japan, and Canada (*6*,*13*–*18*), accompanied by increasing cases among women of reproductive age (*6*,*13*,*15*,*17*–*19*) and cases of congenital syphilis (*6*,*13*,*16*,*17*,*19*,*20*). In the United States, the rate of congenital syphilis increased annually since 2013, increasing 8-fold to 77.9/100,000 live births by 2021 (*17*,*19*). In that year, 46 states and the District of Columbia reported >1 cases of congenital syphilis (*17*).

In Australia, the rate of infectious syphilis cases increased by 316%, from 5.0/100,000 population in 2010 to 20.8/100,000 population in 2020 (*21*). In 2011, an outbreak mainly affecting Indigenous Australians began in northeastern Australia (*22*) and then migrated across the north and into northern Western Australia in 2014–2016 (*22*,*23*). Starting in 2012, cases of infectious syphilis increased in major cities, initially among men and, since 2015, among women. The rate of infectious syphilis cases among men in Australia increased by 36% from 2016 to 2020, and the rate among women of reproductive age rose by 109% (*15*).

We describe the changing demographics among the rising cases of infectious syphilis in Perth, Western Australia, Australia, and the resulting local policy initiatives implemented to reduce the risk for congenital syphilis, including the preliminary outcomes of a holistic multiagency antenatal program for pregnant women with syphilis. This information could be applied in other jurisdictions facing a resurgence of syphilis, particularly those with similar socioeconomic profiles.

## Setting

Perth, population ≈2.1 million, is the capital city of the state of Western Australia, located on the southwestern coast of Australia. The city is culturally diverse; 2% of residents are Indigenous Australians, 60.9% of residents have >1 parent born overseas, 40.5% of residents were born overseas, and a non-English language is used in 23.7% of households. The median weekly household income in Perth (Australian dollars) is $1,865, which is above the national equivalent ($1,746) (*24*).

In Western Australia, under Public Health Act 2016, syphilis of all stages and congenital syphilis are notifiable to the Western Australia Department of Health by the medical or nurse practitioner attending the patient and the reporting laboratory (*25*). Those notifications are then assigned to the local public health unit for follow-up and action.

## Definitions

In Australia, the term culturally and linguistically diverse (CALD) is used to describe persons and populations with particular cultural or linguistic affiliations. The definition often differs according to the indicators collected by the reporting organization (*26*) but may include characteristics such as country of birth, spoken English proficiency, and main language spoken at home (*27*). The term Indigenous Australians describes Aboriginal people (descendants from the original inhabitants of Australia) and Torres Strait Islander people (from the Torres Strait Islands, located northeast of Australia). In Western Australia, the descendants of the original inhabitants are Aboriginal people.

Infectious syphilis encompasses primary, secondary, early latent, and probable infectious syphilis. Those stages cover up to 2 years from the putative time of acquisition (*2*).

## Data Sources

With approval from the corresponding data custodians, we obtained data on syphilis in Perth for 2001–2021 from the Western Australian Notifiable Infectious Diseases Database. We calculated rates by using the Australian Bureau of Statistics census-derived population data from the Epidemiology Branch within the Western Australia Department of Health. We extracted data about pregnant women and persons experiencing homelessness with syphilis from the Metropolitan Communicable Disease Control (MCDC) Syphilis Register.

## Contextualizing the Policy

**Figure 1:**
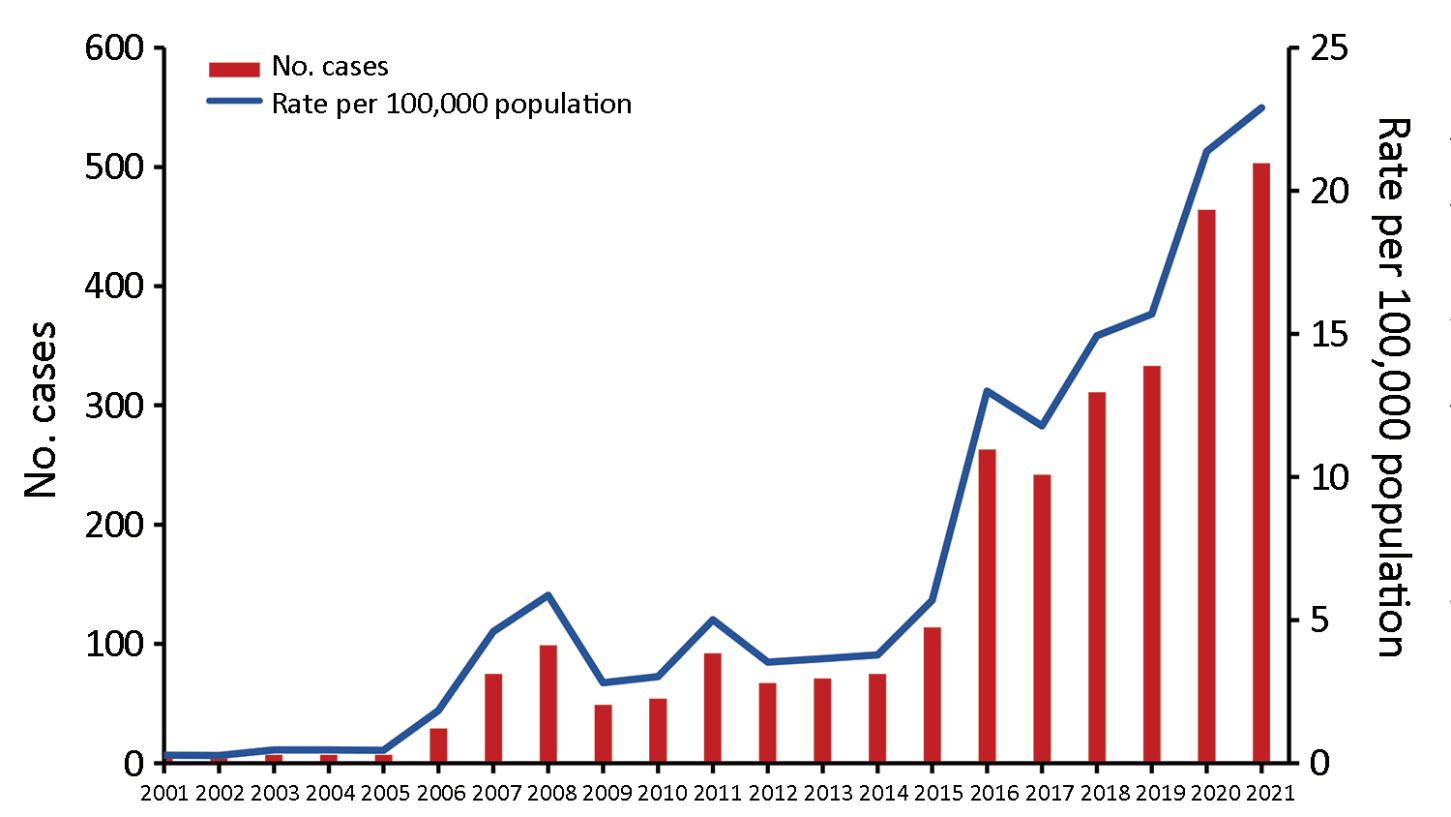
Infectious syphilis cases over time, Perth, Western Australia, Australia, 2001–2021. Numbers of cases were obtained from the Western Australian Notifiable Infectious Diseases Database, Department of Health Western Australia (January 2022); rates were calculated from the Australian Bureau of Statistics census-derived population data from the Epidemiology Branch, Public and Aboriginal Health Division, Western Australia Department of Health (February 2023).

In Perth, the number and rate of infectious syphilis cases have risen substantially, particularly since 2015, reaching 22.9 cases/100,000 population in 2021 (Figure 1). From 2001 through 2005, there were very few cases; the rate remained <0.5/100,000 population. In 2006, the rate increased to 1.8/100,000 population and fluctuated over the next decade, reaching 5.7/100,000 population in 2015; in this period, cases were predominantly among men who have sex with men. From 2015 through 2021, cases of infectious syphilis surged by 312%, coinciding with spread to the heterosexual population, outbreaks in northern Australia, the introduction and rising use of dating apps, and reports of transactional sex for illicit drugs or accommodation. Cases continued to increase despite various COVID-19 pandemic restrictions (*13*), as has been observed elsewhere (*17*,*18*).

**Figure 2:**
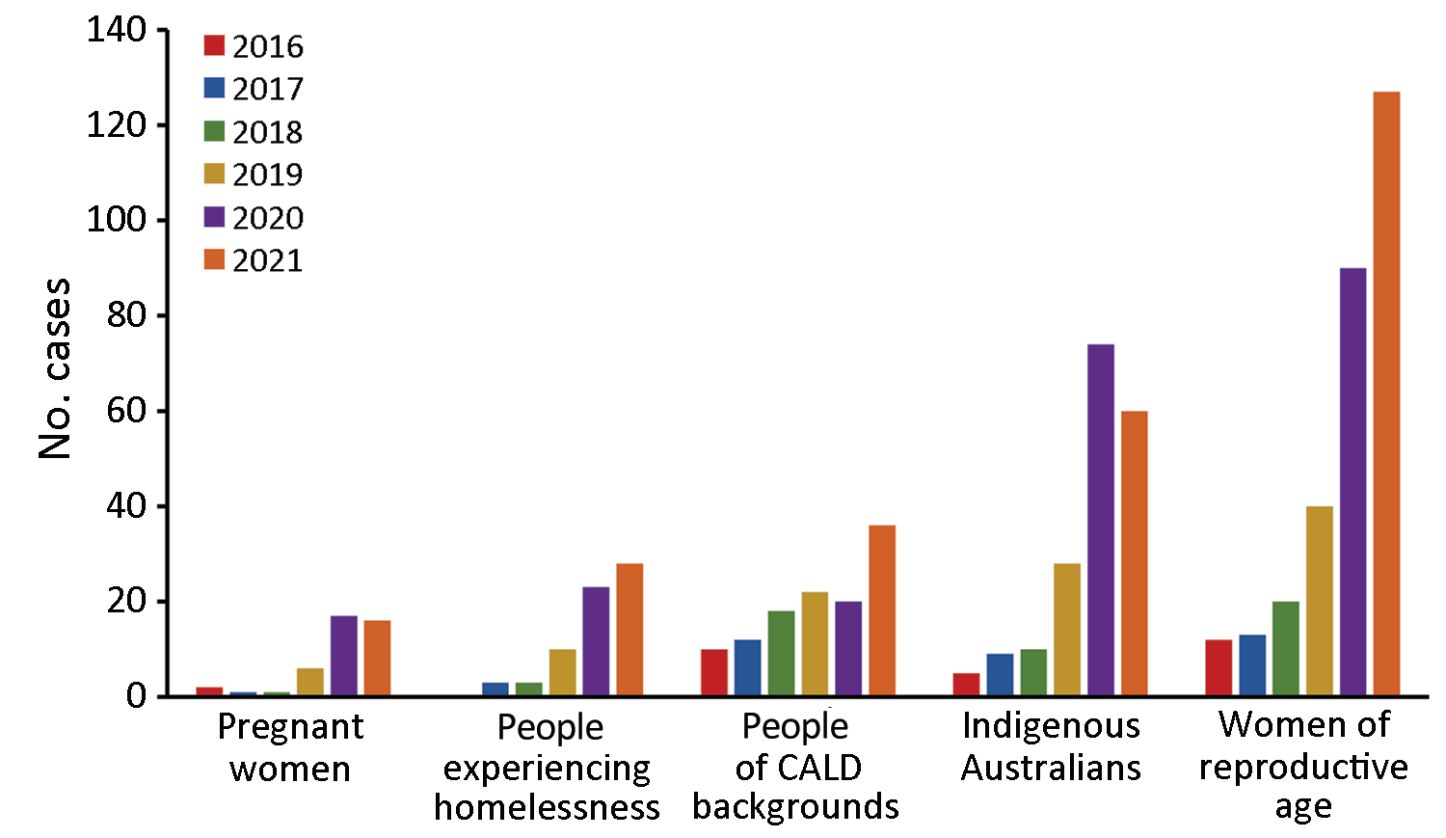
Number of infectious syphilis cases among pregnant women, persons experiencing homelessness, persons of CALD backgrounds, Indigenous Australians, and women of reproductive age, Perth, Western Australia, Australia, 2016–2021. Categories are not mutually exclusive (e.g., a person may fall into >1 category). Data obtained from the Western Australian Notifiable Infectious Diseases Database, Department of Health Western Australia (January 2022) and the Metropolitan Communicable Disease Control Syphilis Register, Metropolitan Communicable Disease Control (January 2022). CALD, culturally and linguistically diverse (persons born in a country other than Australia and who speak a non-English language at home).

Case escalation since 2015 has been accompanied by a rise in the number and proportion of cases among priority populations, including women of reproductive age and the subset who are pregnant, persons experiencing homelessness, persons of CALD backgrounds, and Indigenous Australians (Figure 2). The proportion of cases among Indigenous Australians has increased considerably, from 1.9% in 2016 to 15.9% in 2020 and to 11.9% in 2021.

For women of reproductive age, cases of infectious syphilis have risen from 2.8 cases/100,000 population in 2016 to 28.7/100,000 population in 2021 population. Likewise, cases among pregnant women in Perth rose 8-fold from 2016 through 2021, and there were 5 cases of congenital syphilis from 2018 through 2021 and only 2 cases in the preceding 10-year period.

## Establishing Priority Areas

In July 2020, the Western Australian Chief Health Officer declared a syphilis outbreak in Perth (*28*), which prompted an organized, cross-agency collaborative effort, including establishing the Metropolitan Syphilis Outbreak Response Team (MSORT), a multiagency team with the primary goals of controlling the outbreak in Perth and preventing congenital syphilis. The response is underpinned by a local action plan (*28*) consisting of 5 priority areas: prevention, education, and community engagement; workforce development; testing, treatment, and contact tracing; surveillance and reporting; and antenatal and postnatal care (Table) (*28*). Although the action plan was informed by the WHO 5 strategic directions in relation to STIs (*12*), the priorities were determined by national initiatives (*29*) and a local consultation process involving stakeholders across health sectors, including government and nongovernment organizations and community health and hospital services (*30*).

**Table:** Local initiatives implemented to tackle the syphilis outbreak in Perth, Western Australia, Australia*

Priority area of the Metropolitan Action Plan (*28*)	Descriptions of initiatives
Prevention, education, and community engagement	Syphilis outbreak campaigns to increase awareness among health professionals and the community, including accessible educational resources that reduce stigma and are guided by cultural considerations
	Education of community organizations and community members (e.g., Indigenous, homeless and CALD persons)
	Education of cases and contacts by MCDC public health nurses, Indigenous health professionals and midwife
	Indigenous health team engagement and outreach with community members and persons experiencing homelessness
	Indigenous health team outreach service collaborates with other agencies (e.g., Indigenous community health and health services for persons experiencing homelessness)
Workforce development	Establishment of MSORT, a multiagency team to lead a coordinated outbreak response
	Diversification of MCDC workforce to address population and workforce needs, with a focus on priority populations, including establishment of Indigenous health, clinical midwifery, and general practitioner roles
	Project officer employed to facilitate the development of contextualized models of care and health promotion materials guided by stakeholder engagement for priority groups, including CALD persons
	Epidemiologist employed to help facilitate enhanced surveillance and reporting
	Public health staff engaging with and delivering education to a wide range of health professionals and services, focusing on specialty services that see high priority and/or atypical cases
	General practitioner delivering education to primary healthcare doctors and nurses
	MCDC midwife providing education to health professionals in maternity services
	Development of educational resources and clinician alerts to inform health professionals
	Establishment of regular multiagency SIP and SAPH case management meetings to oversee management of these priority groups
Testing, treatment and contact tracing	Template letter about syphilis treatment, partner notification and repeat testing sent to the test requesting health professional
	Evidence from the congenital syphilis case reviews informed adoption of 3-test routine screening of all pregnant women
	Frontline services engaged in case management and supporting contact tracing
	Engagement with laboratories to improve timeliness of results, particularly for antenatal requests, and changes to reporting algorithms to prevent missed notifications and diagnoses
	Improved processes for ordering parallel testing when monitoring RPRs in pregnant women
	Systematic approach to obtaining maternal and infant syphilis results through a reporting protocol to the MCDC SIP team from hospital obstetric services
Surveillance and reporting	Electronic syphilis public health management register developed to meet the needs of a syphilis outbreak, which has enabled better monitoring and identification of priority populations, including testing, treatment, and contact tracing efforts
	The register is used to generate automated reports (e.g., quarterly reports, individual summaries for case management meetings, and synoptic reports [neonatal management plans])
	Automated alerts remind MCDC staff to confirm receipt of treatment or repeat testing for priority cases
Antenatal and postnatal care	Local STI, antenatal and obstetric guidelines changed to recommend syphilis screening for all pregnant women at initial visit, 28 weeks, and 36 weeks of gestation (or birth if earlier)
	Proactive public health management of pregnant women with syphilis and their sexual contacts
	Monthly multiagency meetings to ensure appropriate care and follow-up of pregnant women with syphilis
	Routine synoptic reporting (neonatal management plans) to guide syphilis testing and management of neonates and their mothers at delivery
	Interagency congenital syphilis case reviews to identify gaps in service delivery and inform service improvement

*CALD, culturally and linguistically diverse; MCDC, Metropolitan Communicable Disease Control; MSORT, Metropolitan Syphilis Outbreak Response Team; RPR, rapid plasma reagin; SAPH, Syphilis Among People Experiencing Homelessness; SIP, Syphilis In Pregnancy; STI, sexually transmitted infection.

MSORT has been pivotal in raising awareness about the outbreak, developing clinician alerts and health promotion materials, educating the workforce, and revising guidelines (*5*,*31*–*34*). Moreover, the local public health unit, MCDC, works closely with local service providers and has helped establish multidisciplinary committees involving primary health, community health services, and hospitals to deliver additional outreach and services for priority groups, including pregnant women, Indigenous Australians, and persons experiencing homelessness.

## Learning from Missed Opportunities

Analyzing congenital syphilis cases can identify opportunities for improved processes and service delivery to prevent future cases. Each case of congenital syphilis is a sentinel event. In Western Australia, all cases are reviewed by a panel consisting of health professionals and other relevant service providers involved in the management of the mother and infant (or fetus, in instances of stillbirth). The purpose of the reviews is not to attribute blame but rather to review the clinical and public health management of the mother and infant to develop recommendations and a plan for service improvement (*30*,*35*). The reviews also provide an opportunity to increase awareness and deliver education (*31*,*35*).

A public health report, which summarized 8 congenital syphilis cases and 1 near miss occurring across the state of Western Australia from January 2019 through June 2021, highlighted that congenital syphilis is generally not attributable to a single factor and encompasses complex social issues, health disparity, access difficulties, and varying levels of healthcare (*31*). Of the neonates with congenital syphilis born to women residing in Perth, the reviews identified pregnant women who tested negative for syphilis at their first antenatal visit but received no repeat syphilis testing during the remainder of their pregnancy, leaving infection acquisition undetected (*31*). In some instances, atypical or subtle clinical manifestations of syphilis remained undetected or misdiagnosed during the antenatal period.

## Dynamic Electronic Syphilis Register

An effective response to any disease is reliant on efficient access to an accurate and comprehensive data surveillance system. To meet the needs of monitoring, reporting, responding to, and managing the syphilis outbreak, MCDC developed an electronic syphilis public health management register. The database can be used to review rapid plasma reagin (RPR) trends, treatment history, and contact tracing status, including identification of reinfections among past case-patients and contacts named on multiple occasions. Data completeness is supported by mandatory notification requirements.

The register has streamlined processes for case management, including a range of automated functions to generate enhanced surveillance reports, case summaries for meetings that include key points of discussion, handover, and neonatal management plans, as well as alerts for repeat testing, which trigger active public health follow-up with the responsible clinician. Furthermore, it enables monitoring of changing demographic profiles, including cases among CALD persons, persons experiencing homelessness, and persons who inject drugs or engage in transactional sex. Because cases among these population groups can be complex, to inform resource allocation, public health staff at MCDC review automated quarterly reports that include trends in demographic profiles.

## Improving Reporting and Timeliness of Syphilis Test Results

The approach to notification of syphilis cases varied by laboratory. Some laboratories reported all positive syphilis test results (even if the RPR was negative), and others reported only according to minimum RPR cutoffs. To improve consistency for women of reproductive age, positive syphilis serology results regardless of the RPR are now reported by all laboratories because cases of early infectious syphilis and untreated early and late latent syphilis were occasionally missed.

Efficient reporting of, and access to, syphilis results help ensure timely case management, which is particularly valuable when managing syphilis in pregnant women, for whom prompt diagnosis, treatment, and contact tracing are pivotal for preventing congenital syphilis. Engaging with local laboratories to improve timeliness from specimen collection to reporting has led to development of an urgent syphilis testing protocol for pregnant women who attend major maternity hospitals and have not accessed regular antenatal care.

Regular parallel RPR monitoring during pregnancy is useful for monitoring treatment response and diagnosing reinfection. In some instances, MCDC staff members identified delays in parallel RPR testing for pregnant women with syphilis. Consultation with local laboratories has led to a revised protocol for this cohort to avoid preventable delays.

## Workforce Development and Community Engagement

Because syphilis is often considered to be a rare condition in Australia (*36*), educating health professionals and the community of its resurgence is critical. Health professionals need to know how to diagnose the disease (clinical features and testing), manage cases and sexual contacts, and follow current guidelines. Local doctors and nurses have delivered education to a wide range of healthcare professionals; efforts have been focused on professionals who receive high-priority or atypical cases, such as dentists and oral surgeons, ophthalmologists, mental health professionals, antenatal service providers, emergency department workers, and general practitioners.

Progressively, local frontline services have become more engaged in opportunistic screening, case management, and contact tracing. For example, opportunistic syphilis screening is now offered to persons entering correctional facilities, and the emergency department at one tertiary hospital has established enhanced screening practices and instigated patient management plans.

Whenever a case of infectious syphilis is notified to MCDC, public health nurses advise healthcare professionals over the phone and by template letter about treatment, partner notification, and repeat testing. When needed, persons with syphilis and their sexual contacts will also be interviewed and counseled. Although effective, those methods are resource intensive and can be difficult to maintain.

The Western Australian Department of Health has developed syphilis campaigns that have raised community awareness of the outbreak. The campaigns aim to reduce STI stigma and are guided by cultural considerations. Educational resources and clinician alerts for health professionals have promoted the “test, treat and trace” message, the value of additional routine testing of pregnant women, and access to culturally guided care (*34*). Outgoing emails from MCDC are accompanied by a signature block with an embedded link to local syphilis guidelines.

To focus on local population needs, the MCDC workforce has been expanded and includes public health doctors and nurses, a general practitioner, a clinical midwife, Indigenous health professionals, an epidemiologist, and a project officer. The team has developed effective collaborations with many local health and community organizations and members. The general practitioner delivers syphilis education to primary physicians and practice nurses. The midwife manages cases in pregnant women, liaising closely with clinicians and providing education to the pregnant women and their sexual contacts. The Indigenous healthcare professionals provide a vital outreach role and help Indigenous Australians and persons experiencing homelessness receive care in culturally safe ways (*37*). They support access to testing and treatment, including transportation assistance, where necessary, and they help to find hard-to-reach case-patients and contacts. The epidemiologist undertakes surveillance and reporting functions, examines the timeliness of treatment delivery, and analyzes the success of contact tracing efforts. The project officer develops models of care, engages with CALD community organizations and members, and provides health promotion materials that are easy to read and consumer focused.

Substantial progress has been made to enhance service delivery and accessibility for high-priority populations affected by syphilis in Perth; however, further actions are needed to ensure accessible care for all. Because of resource constraints amid a pandemic, work has only recently commenced to enhance service delivery for CALD populations (*28*). Ongoing work will be required to ensure that service provision continues to align with community needs.

## Expanding Routine Pregnancy Screening

The WHO STI guideline (*38*), Centers for Disease Control and Prevention STI guidelines (*39*), and Australian Government pregnancy care guidelines (*23*) recommend screening all pregnant women for syphilis at their first antenatal visit. To date, recommendations regarding additional testing have been based on risk (depending on the woman’s demographic profile) and background local syphilis epidemiology (*23*,*39*). More recently, data have demonstrated that testing only 1 time (during early pregnancy) may result in missed cases in which maternal syphilis is acquired later in gestation, particularly in areas where incidence of syphilis is increasing (*8*,*9*,*40*).

Risk factors can be challenging or absent, and cases may be missed if risk factors are relied on to guide testing. Because of the complexities that surround identifying risk factors (*41*) and findings from local congenital syphilis reviews, local STI, antenatal, and obstetric guidelines now recommend screening all pregnant women in Perth at their initial visit, at 28 weeks, and at 36 weeks of gestation (or delivery, if earlier) (*5*,*30*,*32*). Three-test screening for syphilis in all pregnant women helps normalize testing, recognizes that pregnant women remain sexually active, mitigates against unrecognized risk factors, and offers an opportunity to detect syphilis in pregnant women who have no or subtle signs and symptoms (*41*). Locally, universal screening at 28 weeks has already helped prevent at least 4 congenital syphilis cases since its introduction during 2021.

## Enhancing Interagency Collaboration

In late 2020, MCDC introduced interagency and multidisciplinary case management meetings for 2 priority populations: Syphilis In Pregnancy (SIP) and Syphilis Among People Experiencing Homelessness. All pregnant women who received a diagnosis of syphilis during pregnancy, have a history of inadequately treated syphilis, or have completed treatment for infectious syphilis within the 12 months preceding pregnancy are monitored by the SIP committee. The monthly meetings are a collaboration between public health professionals, sexual health physicians, an infectious diseases pediatrician, a neonatologist, midwives, an obstetrician, Indigenous health services, homeless health services, and health professionals from the Department of Justice when needed. Items considered are treatment decisions, access issues, risk assessment of the fetus, neonatal management plans, and contact tracing. This monthly collaboration enables timely support while maximizing the use of finite resources.

## Supporting Optimal Management during Delivery

Early on, the SIP committee found that guidelines (*5*,*42*) for investigating and managing deliveries were not always followed, an issue that has also been described in other jurisdictions in Australia (*14*,*43*). That finding led to development of neonatal management plans, which are synoptic reports generated from the Syphilis Register with data for pregnant women with syphilis at ≈32–34 weeks of gestation (including demographics, stage of syphilis, results, treatment, contact tracing, and level of risk). Based on the data, a risk category (no, low- or high-risk for congenital syphilis) is assigned at the appropriate monthly meeting. Recommended maternal and neonatal investigations and treatment are based on risk category, and contact details of specialist services are provided (*5*,*42*). To guide management at delivery, the neonatal management plan is filed in the women’s maternity hospital record before 36 weeks of gestation and discussed with the woman at an antenatal appointment.

Those plans have been well received by local maternity units and are now actively sought for pregnant women with syphilis who deliver in Perth. Hospital obstetric services report back to the MCDC SIP team, providing feedback on maternal and infant syphilis testing, information about the clinical examination of the neonate, and confirmation about any relevant treatment given to the infant.

## Resourcing

Existing resources were used to support initiatives where possible. However, public health personnel developed a detailed business case and submitted it to the Western Australia Department of Treasury to expand the workforce, including at MCDC. Although successful, funding was limited to an initial 2-year period. In addition, MCDC received a modest 1-time grant for workforce development, which enabled increased education and outreach to clinicians and to Indigenous health and CALD service providers.

## Preliminary Outcomes

From January 1, 2021, through September 30, 2022, the SIP committee monitored 63 pregnant women to the time of delivery or transfer to another health service region. No woman in the program delivered an infant with congenital syphilis.

Over the same period, 49 neonatal management plans were prepared as the program was consolidated. Of the 39 plans recommending infant treatment and mother/infant investigation at delivery (low- or high-risk plans), investigation recommendations were followed for 29 mother/infant pairs (74%), and treatment recommendations were followed for 36 (92%) infants. For a cohort in the Northern Territory of Australia, a similar risk-based approach to neonatal management has been implemented, although without formal generation of a synoptic report, which identified that only 52% of at-risk neonates received appropriate testing and 42% received adequate treatment at birth (*43*).

The program has enabled better monitoring of other health issues during pregnancy because of high attendance at antenatal appointments and, where required, has helped link women to social and community support for nonhealth issues, including housing. The holistic multiagency nature of this program, along with increased antenatal screening, community engagement with priority populations and key organizations, and strengthened collaboration with frontline services, have contributed to the positive outcomes.

## Conclusions

The re-emergence of syphilis in Perth, and in locations in other high-income countries, has been accompanied by increasing case diversity and substantial involvement of women of reproductive age. Multilevel coordinated action that aligns with population needs is required to address this re-emerging disease effectively. Key elements include interagency collaboration, community engagement, workforce education, enhanced screening, ready access to treatment, contact tracing support, and surveillance and reporting. As rates of syphilis across the world increase, we urge other jurisdictions experiencing similar caseloads to consider ways to reduce cases of congenital syphilis. Moreover, given that adherence to management guidelines in the evaluation of mother/infant pairs at delivery is suboptimal, implementing structured neonatal management plans can support optimal evidence-based care at birth. In our setting, the efforts that were successful in preventing additional cases of congenital syphilis were conducting multiagency meetings for managing pregnant women with syphilis, using a dynamic electronic syphilis register, adopting synoptic reporting to guide management at delivery, undertaking community outreach and engagement with health services, and increasing routine syphilis screening during pregnancy.
